# Efficacy of medication therapy for patients with chronic kidney disease and heart failure with preserved ejection fraction: a systematic review and meta-analysis

**DOI:** 10.1007/s11255-021-03025-z

**Published:** 2021-10-20

**Authors:** Lei Yang, Nan Ye, Weijing Bian, Hong Cheng

**Affiliations:** Beijing Anzhen Hospital, Capital Medical University, No.2 Anzhen Street, Chaoyang District, Beijing, 100029 People’s Republic of China

**Keywords:** Chronic kidney disease, Heart failure with preserved ejection fraction, Medication therapy, Meta-analysis, Systematic review

## Abstract

**Background:**

The prevalence and mortality of heart failure with preserved ejection fraction (HFpEF) are high in patients with chronic kidney disease (CKD). However, there is still a lack of recommendations for the medication therapy of these patients in the guideline so far.

**Methods:**

We conducted a systematic review and meta-analysis of all the studies assessing medication therapy for patients with CKD and HFpEF by July 21, 2021. Pooled analysis was performed using a random-effect model and the quality assessment was performed. In our research, we followed to the Preferred Items for Systematic Reviews and Meta-Analyses (PRISMA) guidelines. The meta-analysis was registered on PROSPERO.

**Results:**

We finally identified six studies, three of which were randomized controlled trials and the others were retrospective cohort studies. The results of meta-analysis including three retrospective cohort studies showed that renin–angiotensin system inhibitors had significantly reduced all-cause mortality by 14% (3 studies, 3816 patients, HR 0.86; 95% CI 0.79–0.95; *I*^2^ = 49%; *P* = 0.003), and all-cause hospitalization by 11% (2 studies, 2350 patients, HR 0.89; 95% CI 0.85–0.94; *I*^2^ = 0%; *P* < 0.00001) in patients with CKD and HFpEF. However, there was no significant reduction in the risk of hospitalization for heart failure (3 studies, 3816 patients, HR 0.88; 95% CI 0.75–1.04; *I*^2^ = 75%; *P* = 0.13). One of the studies focused on the sacubitril–valsartan showed that sacubitril–valsartan was associated with a reduced risk of hospitalization for heart failure and cardiovascular death (RR 0.79, 95% CI 0.66–0.95). The study focused on the carvedilol did not show a significant reduction in the risk of hospitalization for heart failure and cardiovascular death (HR 0.917, 95% CI 0.501–1.678).

**Conclusions:**

For patients with CKD and HFpEF, renin–angiotensin system inhibitors is associated with significant benefits in all-cause mortality and all-cause hospitalization but has no significant effect on hospitalization for heart failure. The subgroup analysis of one RCT study focused on ARNI showed that although long-term treatment with sacubitril–valsartan may reduce the risk of hospitalization for heart failure and cardiovascular death, more studies are needed to confirm that.

**Supplementary Information:**

The online version contains supplementary material available at 10.1007/s11255-021-03025-z.

## Introduction

Chronic kidney disease (CKD) is a common chronic disease that has a high prevalence, especially in developing countries [1, 2]. An epidemiological study in 2012 conducted in China showed that the morbidity of CKD can be as high as 10.8% [3]. Compared with healthy people, patients with CKD are more likely to have cardiovascular disease and the morbidity of heart failure (HF) is higher. Studies have shown that 30% of patients with CKD were complicated with HF [4], and the morbidity of HF was 17–21% [5]. In China, 16.02% of patients with CKD are complicated with HF [6]. In 2016, the European Society of Cardiology guidelines for the diagnosis and treatment of acute and chronic heart failure (ESC) firstly divided HF into three categories according to ejection fraction: heart failure with reduced ejection fraction (HFrEF), heart failure with intermediate ejection fraction (HFmrEF), and heart failure with preserved ejection fraction (HFpEF), which made HFpEF get more attention. HFpEF is an important subtype of heart failure, which mainly characterized by the presence of symptoms and signs of HF, but with an ejection fraction ≥ 50% [7]. In patients with HF, HFpEF account for more than 40% [8, 9]. Compared with HFrEF, HFpEF has its own characteristics, including most of them are women, more likely to be complicated with other diseases, such as obesity, renal insufficiency, diabetes mellitus, atrial fibrillation, etc. In the above complications, the most common complication is CKD [10, 11]. At the same time, HFpEF is the most common type of HF in patients with CKD [12]. In addition to a lot of same comorbidities in two diseases, another mechanism of coexistence of CKD and HFpEF is the concurrent macrovascular lesions caused by CKD and the direct effects of some renal factors on the heart or microvessels [13].

Patients with CKD and HFpEF have high all-cause mortality [14–16], and it increases with the decrease of estimated glomerular filtration. Renal dysfunction can aggravate the retention of water and sodium in patients with HF, which will result in a further increase in volume overload. A multicenter prospective cohort study showed that reduced glomerular filtration rates were associated with mortality in patients with HFpEF [17]. Another study showed that renal insufficiency was independently associated with abnormal cardiac mechanics in patients with HFpEF, leading to adverse outcomes [16]. Therefore, it is necessary to know how to treat patients with CKD and HFpEF to reduce the readmission rate and mortality of patients.

However, most of the medication therapy which has evidence-based support is mainly for patients with HFrEF. There are few studies on the treatment of HFpEF, and there are no consistent conclusions either. Therefore, there is no high-level recommendation of medication therapy for this group of people in the guidelines. For HFpEF, 2013 foundation of the American college of cardiology (ACCF)/American heart association (AHA) Guideline for the Management of Heart Failure, and 2016 ESC guidelines put forward the main medication therapy is only treat the complications (such as high blood pressure) and using diuretics to alleviate symptoms and signs caused by volume overload [7, 18]. The medication therapy for patients with CKD and HFpEF was not mentioned at all.

In recent years, some clinical trials about medication therapy for patients with CKD and HFpEF have occurred, including the “golden Triangle” medication which is recognized for the treatment of HFrEF: β-blockers, renin–angiotensin system (RAS) inhibitors, aldosterone receptor antagonist, as well as the research hotspot—sacubitril–valsartan (ARNI) and so on. However, its conclusions are not entirely consistent. In our study, a systematic review and meta-analysis were conducted to explore the effect of medication therapy for patients with CKD and HFpEF.

## Materials and methods

### Data sources and searches

**Fig. 1:**
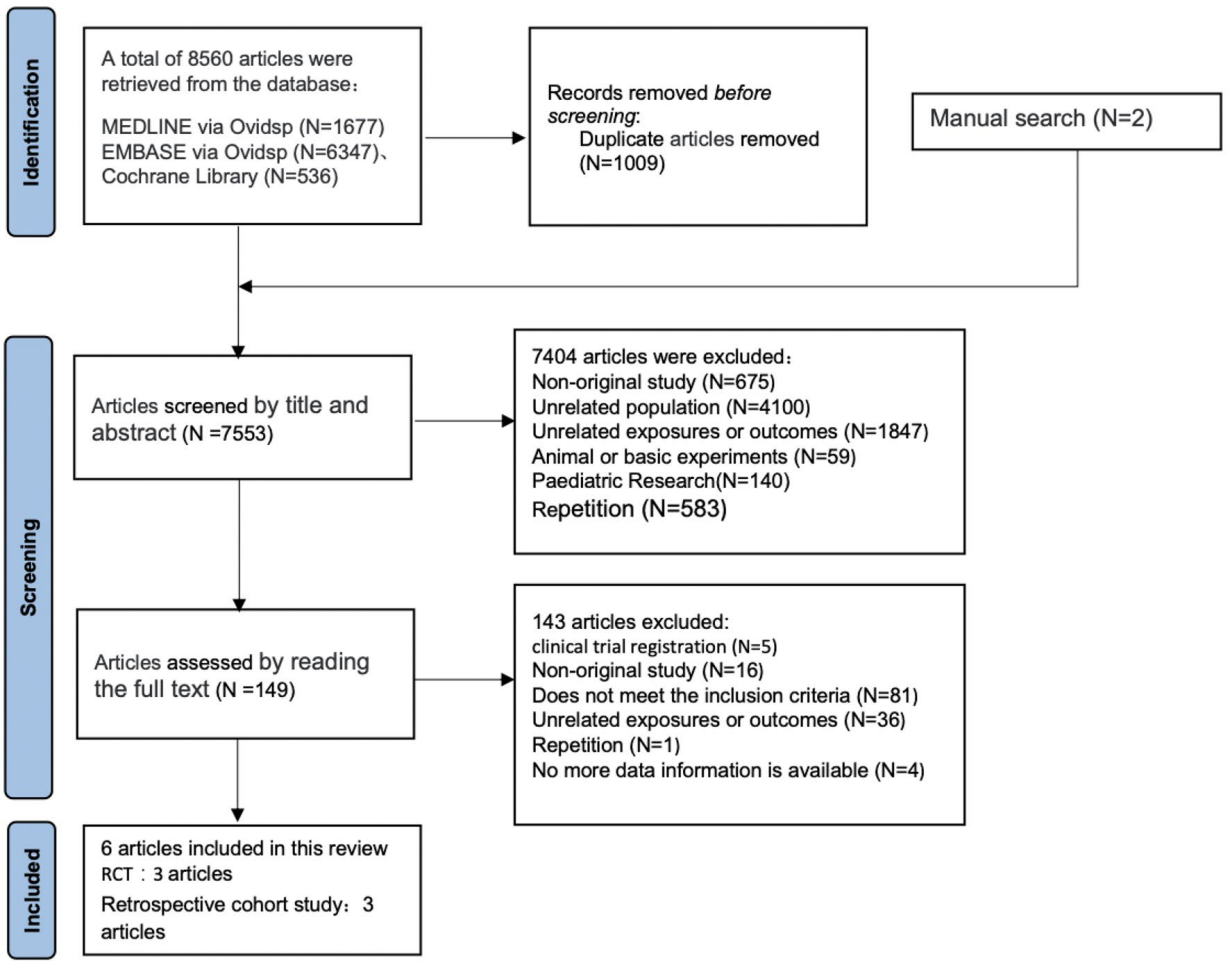
Screening process followed to PRISMA 2020 flow diagram

All types of literature about medication therapy for patients with CKD and HFpEF were searched by July 21, 2021, from relevant bibliographic databases, including MEDLINE via Ovidsp (1946—present), EMBASE via Ovidsp (1947—present), and the Cochrane Library. It contains reviews, meetings, and studies published in the ClinicalTrials.gov website. The retrieval strategy is in the supplementary material. Also, we manually searched the reference lists of eligible studies and clinical trials which are referred to in academic conference. In addition, unpublished studies were sought in references of all selected studies, relevant conference abstracts, and from the ClinicalTrials.gov website. There were no language or region selection restrictions. The specific screening process was followed PRISMA 2020 flow and it is shown in Fig. 1.

### Study outcomes

The primary outcome was all-cause mortality. Secondary outcomes were all-cause hospitalization, hospitalization for heart failure, cardiovascular death, the change of biomarkers (NT-proBNP, BNP), or composite endpoint of hospitalization for heart failure and cardiovascular death.

### Study screening

The process of screening study was independently completed by two reviewers. After the preliminary screening by viewing the abstracts, the full text of selected articles will be read carefully to determine whether the study should be included or not. Any disagreement on the eligibility of a study was handled by a senior reviewer in the team. Maintain a complete record of the decision-making process.

### Data extraction and quality assessment

Data of each study were extracted independently by two reviewers (LY and NY), including publication time, study’s design, countries participants from, criteria of inclusion and exclusion, the definition of HFpEF and CKD, follow-up duration, sample sizes, baseline characteristics, interventions, outcomes, and elements used to assess the quality. For the missing data, we contacted the authors through email and Internet as far as possible to get.

The Cochrane risk-of-bias tool [19] was used to assess the risk of bias for randomized controlled trials (RCTs). The Risk of Bias in Nonrandomized studies of Interventions (ROBINS-I) tool [20] was used to assess the risk of bias for observational studies. At the same time, Newcastle–Ottawa scale (NOS) [21] and National Heart, Lung, and Blood Institute (NHLBI) [22] were used to assess the risk of bias for observational studies, too. In addition, we add the risk of bias associated with different criteria for HFpEF and CKD: those conformed to the definition of HFpEF in 2016 ESC guidelines [7] are considered as “low risk”, those did not conform to the definition are considered as “high risk” and those did not describe in the article are considerate as “not clear”, similarly, those conformed to the definition of CKD in Kidney Disease: Improving Global Outcomes (KDIGO) [23] are considered as “low risk”, those did not conform to the definition are considered as “high risk” and those did not describe in the article are considered as “not clear”. The publication bias was assessed using a funnel plot and Egger’s Test. The assessment was done at both the study and outcome level. The risk of bias was assessed independently by two reviewers (LY and NY), with a third senior reviewer (HC) handling the conflicting items to determine the level of risk.

### Data synthesis and analysis

We synthesized data from the results of the same medication and a part of the baseline characteristics of the participants taken RAS inhibitors. Hazard ratio (HR) and 95% confidence interval calculation of standard error (SE) were calculated for data synthesis of survival analysis variables. The sample size of experimental and control group and the corresponding number of events were used for data synthesis of dichotomous variables. The average (Mean) and standard deviation (SD) were used for data synthesis of continuous variables. The random effects model was used for all three variables. *I*^2^ was used to assess the statistical heterogeneity of the combined results. *P* < 0.05 was defined as statistically significant. Stata 15.1 and Review Manager 5.3 were used to perform the above statistical analysis process.

## Results

### Included studies and characteristics

In all six studies included after screening, three were RCTs (one study focused on carvedilol [24], two studies focused on sacubitril–valsartan [25, 26]), the others were retrospective cohort studies (all studies focused on RAS inhibitors [27–29]). The three studies focused on RAS inhibitors were included in the meta-analysis. Basic information and important characteristics of each study are listed in Table 1. The average age of the study population was 71.1–83.0 years. Most of the study population were female, accounting for more than 50%, atrial fibrillation accounted for 29.3–47.2%, and hypertension accounted for 78.0–95.6%.

**Table 1:** Characteristics of included studies

Study	Experimental/control	Design type	*N*	Age (year)	Female (%)	BMI (m/kg^2^)	Rate (times/min)	SBP (mmHg)	DBP (mmHg)	LVEF (%)	NYHA class I/II (%)	NYHA class III/IV (%)	eGFR (mL/min per 1·73 m^2^)	SCr (mg/dl)	AF (%)	HT (%)	DM (%)	Myocardial infarction (%)	Follow-up (year)
Solomon 2012*	Sacubitril–valsartan/valsartan	RCT	301	71.1	56.5	29.9	69.5	136.3^C^	78.4^C^	58	80.0	20.0	65.5	NA	41.5	93.7	37.9	20.6	0.7^a^
Solomon 2019*	Sacubitril–valsartan/valsartan	RCT	4796	72.7	51.7	30.2	70.5	130.5	NA	57.6	80.1	19.8	62.5	1.1	32.4	95.6	43.0	22.6	2.9^b^
Yamamoto 2013	Carvedilol/without carvedilol	RCT	245	80.0	42.0	24.1	73.0	133.5	74.5	62.5	90.6	9.4	58.2	1.0	33.9	80.4	30.6	NA	3.2
Ahmed 2013	RAS blocker/without RAS blocker	Retrospective cohort study	1340	79.0	71.0	NA	NA	154.9	78.6	56	NA	NA	NA	NA	29.3	78.0	44.1	18.4	8^a^
Gurwitz 2017	RAS blocker/without RAS blocker	Retrospective cohort study	1010	83.0	55.8	NA	NA	NA	NA	NA	NA	NA	NA	NA	47.2	79.2	18.4	6.8	1.1^b^
Tsujimoto 2018	RAS blocker/without RAS blocker	Retrospective cohort study	1466	71.6	58.0	32.6	NA	128.3	73.5	NA	62.1	37.9	52.2	NA	41.2	92.4	41.5	26.8	2.8

*BMI* body mass index, *RCT* randomized controlled trial, *SBP* systolic blood pressure, *DBP* diastolic blood pressure, *LVEF* left ventricular ejection fraction, *eGFR* estimated glomerular filtration rate, *SCr* serum creatinine, *AF* atrial fibrillation, *HT* hypertension, *DM* diabetes

The data listed are averages or percentages

^*^Since the baseline data of CKD subgroup was not provided in this paper, the data listed are the original population data in this paper

^a^The article referred to follow-up time, non-mean and median

^b^Median follow-up time

^c^The original data are the median, and the average value is estimated by the conversion formula. Conversion formulas are derived from the literature: Luo et al. [30]

### All-cause mortality

**Fig. 2:**
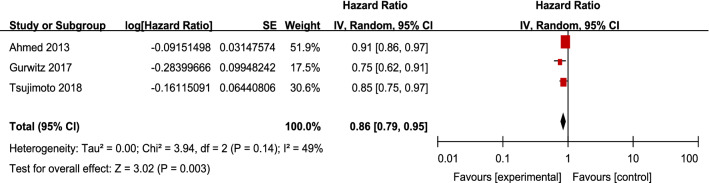
Effects of renin–angiotensin system inhibitors on all-cause mortality

All-cause mortality was reported in three studies [27–29]. For patients with CKD and HFpEF, RAS inhibitors significantly reduced all-cause mortality by 14% (3 studies, 3816 patients, HR 0.86; 95% CI 0.79–0.95; *I*^2^ = 49%; *P* = 0.003). The heterogeneity between studies was low (Fig. 2).

### All-cause hospitalization

**Fig. 3:**
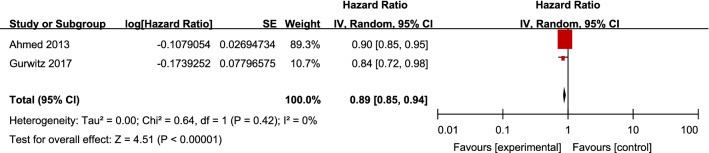
Effects of renin–angiotensin system inhibitors on all-cause hospitalization

All-cause hospitalization was reported in two studies [27, 28]. For patients with CKD and HFpEF, the use of RAS inhibitors resulted in a significant reduction by 11% in all-cause hospitalization (2 studies, 2350 patients, HR 0.89; 95% CI 0.85–0.94; *I*^2^ = 0%; *P* < 0.00001). The heterogeneity between studies was low (Fig. 3).

### Hospitalization for heart failure

**Fig. 4:**
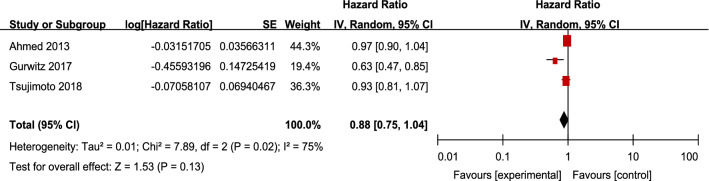
Effects of renin–angiotensin system inhibitors on hospitalization for heart failure

Hospitalization for heart failure was reported in three studies [27–29]. For patients with CKD and HFpEF, RAS inhibitors tended to reduce hospitalization for heart failure, but had no statistical significance (3 studies, 3816 patients, HR 0.88; 95% CI 0.75–1.04; *I*^2^ = 75%; *P* = 0.13). The heterogeneity between studies was high (Fig. 4).

### Baseline characteristics of patients with CKD and HFpEF treated with renin–angiotensin system inhibitors

**Fig. 5:**
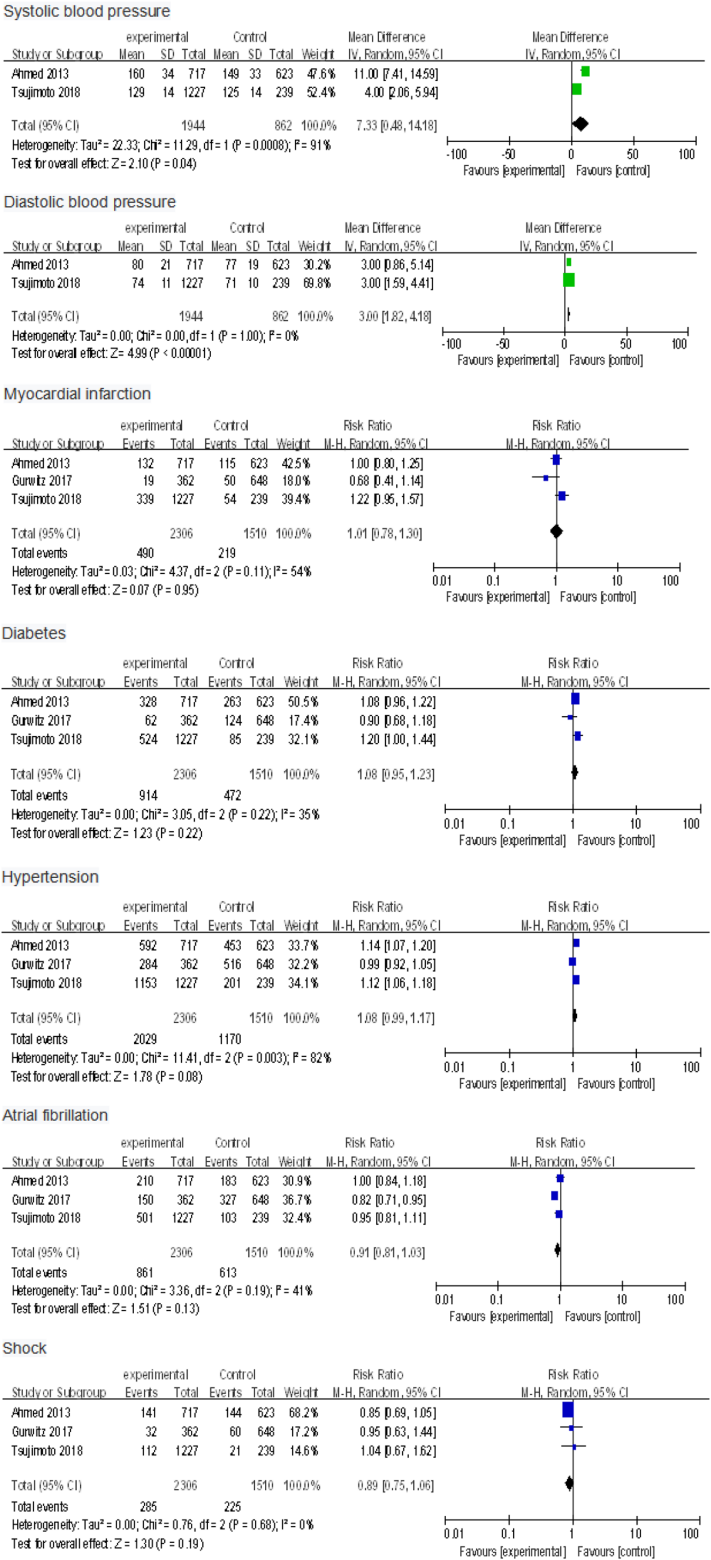
Effects of systolic blood pressure, diastolic blood pressure, history of myocardial infarction, diabetes, hypertension, atrial fibrillation, and shock on the prescription of renin–angiotensin system inhibitors

To explore which patients with CKD and HFpEF in clinic were more likely to be prescribed RAS inhibitors, we synthesized some significant baseline data from experimental and control groups in three studies about this drug. Among them, higher systolic blood pressure (2 studies [27, 29], 2806 patients, MD7.33; 95% CI 0.48–14.18; *I*^2^ = 91%; *P* = 0.04) and diastolic blood pressure (2 studies [27, 29], 2806 patients, MD 3; 95% CI 1.82–4.18; *I*^2^ = 0%; *P* < 0.00001) were more likely to be treated with RAS inhibitors, with statistically significant difference. People combined with myocardial infarction (3 studies [27–29], 3816 patients, RR 1.01; 95% CI 0.78–1.30; *I*^2^ = 54%; *P* = 0.95), diabetes mellitus (3 studies [27–29], 3816 patients, RR 1.08; 95% CI 0.95–1.23; *I*^2^ = 35%; *P* = 0.22), hypertension (3 studies [27–29], 3816 patients, RR 1.08; 95% CI 0.99–1.17; *I*^2^ = 82%; *P* = 0.08), atrial fibrillation (3 studies [27–29], 3816 patients, RR 0.91; 95% CI 0.81–1.03; *I*^2^ = 41%; *P* = 0.13) and shock (3 studies [27–29], 3816 patients, RR 0.89; 95% CI 0.75–1.06; *I*^2^ = 0%; *P* = 0.19) showed no significant difference in their susceptibility of being treated with RAS inhibitors, compared with people without these diseases (Fig. 5).

### Quality assessment

**Fig. 6:**
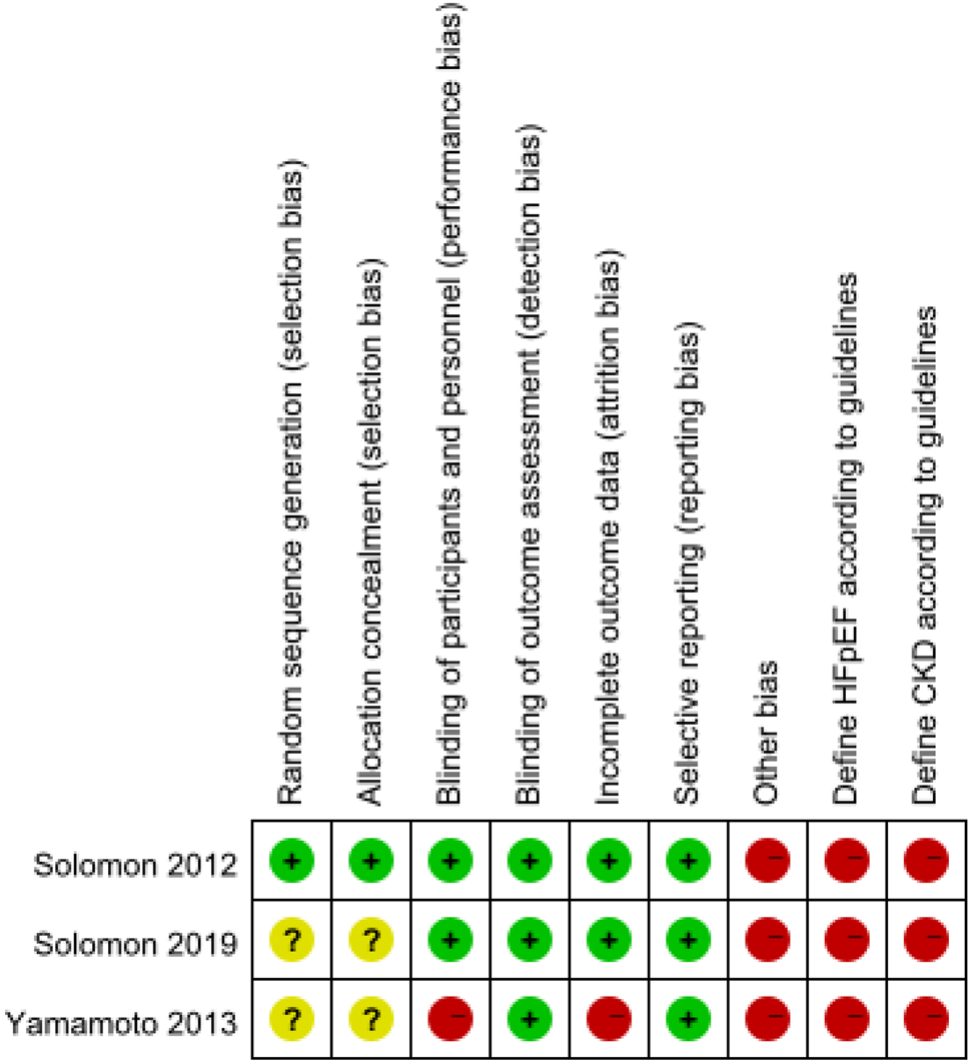
Risk of bias summary for included randomized controlled study

Three RCTs were evaluated using the Cochrane Risk-of-bias tool, and the results are shown in Fig. 6, S1: two studies [24, 25] did not explain specific methods for the generation and distribution of random sequences. In one study [24], the total number of enrolled people, the dose of drugs used, and the incidence of the primary endpoint did not meet the expected standards set before the experiment; the subjects and researchers were not blinded, and the situation of the lost follow-up population was not described. One study [25] did not have specific effect values for CKD subgroup analysis, and excluded the enrolled population who could not tolerate the use of experimental drugs in the transition period for various reasons, which may lead to selection bias. None of the three studies defined HFpEF according to the criteria for heart failure classification of 2016 ESC guidelines, which was defined as “left ventricular ejection fraction ≥ 50%”, and all of them excluded people with glomerular filtration rate less than 30 ml/min/1.73 m^2^. What’s more, because the three randomized controlled studies were all subgroup analyses, the sample size of this subgroup was small. Overall, RCTs were of moderate quality.

**Fig. 7:**
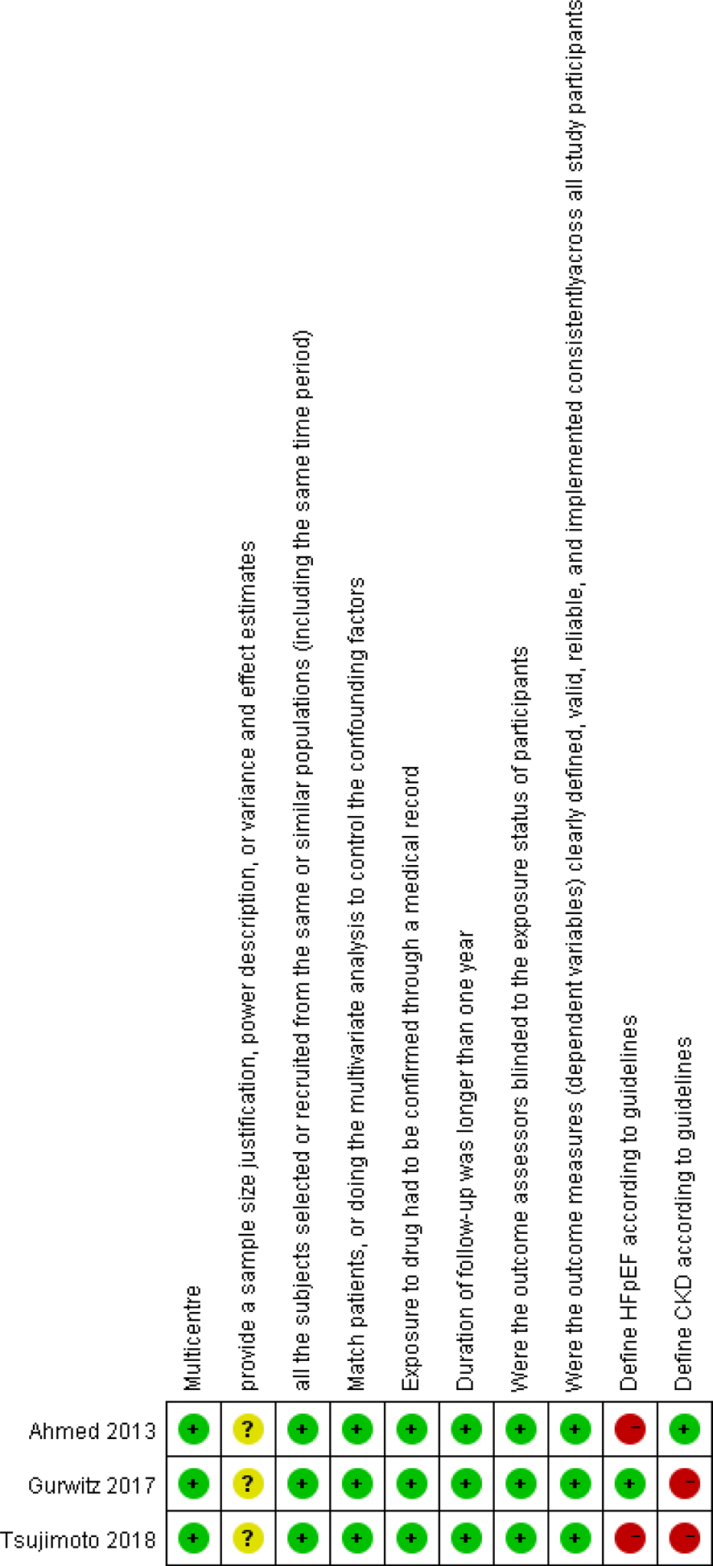
Risk of bias summary for included retrospective cohort study

Three retrospective cohort studies were evaluated by two tools. The results from ROBINS-I was reported in our supplementary materials (Table S1), and the results from NOS and the Quality Assessment Tool from NHLBI are shown in Fig. 7, S2: two studies [27, 29] did not identify HFpEF according to the criteria for heart failure classification of 2016 ESC guidelines, which was defined as “left ventricular ejection fraction ≥ 50%”; one study [29] excluded the population with glomerular filtration rate less than 30 ml/min/1.73 m^2^. One study [28] excluded dialysis patients; none of the three studies described the estimated size of the sample and the estimation of the effect size. In general, retrospective cohort studies were of high quality. The funnel plots for publication bias were shown in Figs. S3–S5. There was a significant publication bias on all-cause hospitalization (*P* < 0.05). No significant publication bias was seen for all-cause mortality (*P* = 0.092) and hospitalization for heart failure (*P* = 0.241).

## Discussion

Our study concluded that RAS inhibitors could significantly reduce all-cause mortality and all-cause hospitalizations in patients with HFpEF and CKD. This may be due to the RAS inhibitors can reduce the pressure of glomerular perfusion, as well as renal burden, and have the function of anti-glomerulosclerosis as well as anti-renal interstitial fibrosis. It also has the functions of dilating blood vessels, lowering blood pressure, reducing retention of water and sodium, reducing cardiac load, so that to reduce myocardial oxygen consumption and improve myocardial ischemia. Therefore, it can improve the prognosis of patients with CKD and HFpEF [31]. However, it should be noted that the population with CKD included in our study is not representative totally. Most of the studies included in the analysis excluded patients with severe renal insufficiency whose eGFR < 30 ml/min/1.73 m^2^. However, the prognosis of this group of patients is poor, and the clinical treatments are limited. The application of RAS inhibitors is more likely to cause adverse events such as hyperkalemia and deterioration of renal function, or lead to adverse outcomes. Therefore, for this part of patients with severe renal insufficiency, more RCTs should be designed to standardize the clinical treatment.

Among the six studies included, except for RAS inhibitors, studies focused on other drugs, which because of lacking the same medication studies or the same endpoints so that data synthesis cannot be conducted, still have important reference significance.

Two RCTs focused on ARNI (PARAMOUNT [26] 和PARAGON-HF [25]) were subgroup analyses of patients with CKD and HFpEF. The population included in PARAMOUNT was EF ≥ 45% and eGFR ≥ 30 ml/min/1.73 m^2^. The experimental and control group were, respectively, received sacubitril–valsartan and valsartan, and the main endpoint was the change rate of NT-proBNP during 12 weeks after taking medicine. The subgroup analysis of CKD showed no significant difference between the treatment and control group. It may be related to the small sample size and short observation period in this study. The PARAGON-HF [25] study conducted later had a larger sample size and longer observation period. The intervention measures and included population of the experimental and control groups were as the same as PARAMOUNT [26] study. The primary endpoint was a composite of hospitalization for heart failure and cardiovascular death. The total time of observation was 4 years. In subgroup of CKD (eGFR < 60 ml/min/1.73 m^2^), compared with control group, experimental group significantly reduced the risk of hospitalization for heart failure and cardiovascular death (RR 0.79, 95% CI 0.66–0.95), while there was no significant difference between the two groups in patients with eGFR ≥ 60 mL/min/1.73 m^2^. Although it is only the result of subgroup analysis, it suggested that long-term treatment of ARNI may improve the cardiovascular prognosis in patients with CKD and HFpEF. The next step is to design more RCTs for this part of population to provide more powerful evidence.

There is only one study [24] focused on carvedilol in the treatment of patients with CKD and HFpEF. This is a subgroup analysis of CKD in RCT, and the average follow-up period was 3.2 years. The population included in the study was EF ≥ 40%, eGFR ≥ 30 mL/min/1.73 m^2^, and patients in the experimental and control groups were treated, respectively, with carvedilol and without carvedilol. Background treatment for both groups was standard cardiovascular therapy without β-blockers. The primary endpoint was composite of hospitalization for heart failure and cardiovascular death. Subgroup analysis based on eGFR showed that experimental group had no significant benefit compared with control group in CKD (eGFR < 60 ml/min/1.73 m^2^) as well as non-CKD (eGFR ≥ 60 ml/min/1.73 m^2^) (HR 0.917, 95% CI 0.501–1.678; HR 0.886, 95% CI 0.356–2.205). Since the total number of people included in this study, the dose of drugs used, and the event rate of the main endpoint did not reach the expected standard set before the study, and the sample size of the subgroup was small, the study and conclusion were limited to some extent, so more studies with large samples and high quality were needed to provide more strong evidence.

CKD with HFpEF is not uncommon in clinical practice, but early clinical diagnosis missed is also a high risk of death. However, medication therapy research for this special population is very limited, and the sample size of the study is also limited. There is a lack of recommendations for treatment, and clinical attention is far from enough. This paper is the first systematic review and meta-analysis on the efficacy of medication therapy for patients with CKD and HFpEF. Our review show that, for patients with CKD and HFpEF, the use of RAS inhibitors can reduce all-cause mortality and hospitalization but has no significant effect on hospitalization for heart failure. However, there are too few studies on promising drugs such as ARNI to draw definitive conclusions, and more research is needed. SGLT2 inhibitor is also a hot research drug. It has been confirmed by successive studies that it can reduce the hospitalization rate and mortality rate of patients with heart failure with reduced ejection fraction and improve the renal prognosis of patients with type 2 diabetes complicated with CKD [32, 33]. Unfortunately, there have been no studies on patients with HFpEF and CKD. Through our analysis and summary, more scientific RCTs can be designed to continuously improve the overall prognosis of patients with CKD and HFpEF.

There are also many flaws in our research. First, the number of studies is small. Due to the particularity of the study population, the study of each medication is less, and even there is only one study for some drugs. What’s more, the synthetic data and meta-analysis of three research are retrospective studies. The lack of high-quality randomized controlled studies, which results in potential confounders and biases, may affect this study. Second, because of the three randomized controlled studies are all subgroup analysis on renal dysfunction, the sample size was small, and the studies were not designed for the endpoint in advance, so the statistical assurance may be insufficient. Third, the included studies lacked the same renal outcomes and adverse events, so the data could not be synthesized and it was difficult to comprehensively evaluate the efficacy and safety of the medication. Finally, due to HFpEF was defined differently in each study, and most studies excluded eGFR < 30 ml/min/1.73 m^2^ or dialysis patients, the resulting selection bias may also affect our final study results.

## Conclusions

The results of this systematic review show that, for patients with CKD and HFpEF, the use of RAS inhibitors can reduce all-cause mortality and hospitalization, but has no significant effect on hospitalization for heart failure. Although the long-term treatment with ARNI is associated with significant benefits on the hospitalization for heart failure and cardiovascular mortality, more studies are needed to confirm that. Carvedilol did not show significant effect in the risk of hospitalization for heart failure and cardiovascular mortality. Due to the lack of research in this area, larger samples and higher quality studies are needed to provide stronger evidence.

Although there are some deficiencies in this systematic review, the conclusions of this study provide some important guidance to clinicians in medication therapy for patients with CKD and HFpEF, also, might provide some direction for the clinical and basic research in this area.

## Supplementary Information

Below is the link to the electronic supplementary material.

Supplementary file1 (DOCX 1159 KB)

## Declarations

### Research involving human and animal participants

This article does not contain any studies with human participants or animals performed by any of the authors.
