# Supplementary material for: Efficacy of medication therapy for patients with chronic kidney disease and heart failure with preserved ejection fraction: a systematic review and meta-analysis
Source: Int Urol Nephrol. 2021 Oct 20;54(6):1435–44. doi: 10.1007/s11255-021-03025-z (PMC9085668; doi:10.1007/s11255-021-03025-z)
Supplement: Supplementary file 1 — Supplementary file1 (DOCX 1159 KB) [file 11255_2021_3025_MOESM1_ESM.docx]

**RISMA 2020 Checklist**

| **Section and Topic** | **Item #** | **Checklist item** | **Location where item is reported** |
| --- | --- | --- | --- |
| **TITLE** | | |  |
| Title | 1 | Identify the report as a systematic review. | Page1 |
| **ABSTRACT** | | |  |
| Abstract | 2 | See the PRISMA 2020 for Abstracts checklist. | Page1 |
| **INTRODUCTION** | | |  |
| Rationale | 3 | Describe the rationale for the review in the context of existing knowledge. | Page1 |
| Objectives | 4 | Provide an explicit statement of the objective(s) or question(s) the review addresses. | Page1 |
| **METHODS** | | |  |
| Eligibility criteria | 5 | Specify the inclusion and exclusion criteria for the review and how studies were grouped for the syntheses. | Page5-6 |
| Information sources | 6 | Specify all databases, registers, websites, organisations, reference lists and other sources searched or consulted to identify studies. Specify the date when each source was last searched or consulted. | Page1,5 |
| Search strategy | 7 | Present the full search strategies for all databases, registers and websites, including any filters and limits used. | Supplementary material |
| Selection process | 8 | Specify the methods used to decide whether a study met the inclusion criteria of the review, including how many reviewers screened each record and each report retrieved, whether they worked independently, and if applicable, details of automation tools used in the process. | Page6 |
| Data collection process | 9 | Specify the methods used to collect data from reports, including how many reviewers collected data from each report, whether they worked independently, any processes for obtaining or confirming data from study investigators, and if applicable, details of automation tools used in the process. | Page6 |
| Data items | 10a | List and define all outcomes for which data were sought. Specify whether all results that were compatible with each outcome domain in each study were sought (e.g. for all measures, time points, analyses), and if not, the methods used to decide which results to collect. | Page6 |
|  | 10b | List and define all other variables for which data were sought (e.g. participant and intervention characteristics, funding sources). Describe any assumptions made about any missing or unclear information. | Page6 |
| Study risk of bias assessment | 11 | Specify the methods used to assess risk of bias in the included studies, including details of the tool(s) used, how many reviewers assessed each study and whether they worked independently, and if applicable, details of automation tools used in the process. | Page6-7 |
| Effect measures | 12 | Specify for each outcome the effect measure(s) (e.g. risk ratio, mean difference) used in the synthesis or presentation of results. | Page7-8 |
| Synthesis methods | 13a | Describe the processes used to decide which studies were eligible for each synthesis (e.g. tabulating the study intervention characteristics and comparing against the planned groups for each synthesis (item #5)). | Page6 |
|  | 13b | Describe any methods required to prepare the data for presentation or synthesis, such as handling of missing summary statistics, or data conversions. | Page7 |
|  | 13c | Describe any methods used to tabulate or visually display results of individual studies and syntheses. | Page7 |
|  | 13d | Describe any methods used to synthesize results and provide a rationale for the choice(s). If meta-analysis was performed, describe the model(s), method(s) to identify the presence and extent of statistical heterogeneity, and software package(s) used. | Page7 |
|  | 13e | Describe any methods used to explore possible causes of heterogeneity among study results (e.g. subgroup analysis, meta-regression). | none |
|  | 13f | Describe any sensitivity analyses conducted to assess robustness of the synthesized results. | Page7 |
| Reporting bias assessment | 14 | Describe any methods used to assess risk of bias due to missing results in a synthesis (arising from reporting biases). | Page7 |
| Certainty assessment | 15 | Describe any methods used to assess certainty (or confidence) in the body of evidence for an outcome. | none |
| **RESULTS** | | |  |
| Study selection | 16a | Describe the results of the search and selection process, from the number of records identified in the search to the number of studies included in the review, ideally using a flow diagram. | Page5-6 |
|  | 16b | Cite studies that might appear to meet the inclusion criteria, but which were excluded, and explain why they were excluded. | Figure1 |
| Study characteristics | 17 | Cite each included study and present its characteristics. | Figure1 |
| Risk of bias in studies | 18 | Page | Page9-10 |
| Results of individual studies | 19 | For all outcomes, present, for each study: (a) summary statistics for each group (where appropriate) and (b) an effect estimate and its precision (e.g. confidence/credible interval), ideally using structured tables or plots. | Page9-10 |
| Results of syntheses | 20a | For each synthesis, briefly summarise the characteristics and risk of bias among contributing studies. | Page 9-10 |
|  | 20b | Present results of all statistical syntheses conducted. If meta-analysis was done, present for each the summary estimate and its precision (e.g. confidence/credible interval) and measures of statistical heterogeneity. If comparing groups, describe the direction of the effect. | Page 8-9 |
|  | 20c | Present results of all investigations of possible causes of heterogeneity among study results. | None |
|  | 20d | Present results of all sensitivity analyses conducted to assess the robustness of the synthesized results. | None |
| Reporting biases | 21 | Present assessments of risk of bias due to missing results (arising from reporting biases) for each synthesis assessed. | Page 10 |
| Certainty of evidence | 22 | Present assessments of certainty (or confidence) in the body of evidence for each outcome assessed. | None |
| **DISCUSSION** | | |  |
| Discussion | 23a | Provide a general interpretation of the results in the context of other evidence. | Page10-13 |
|  | 23b | Discuss any limitations of the evidence included in the review. | Page12-13 |
|  | 23c | Discuss any limitations of the review processes used. | Page12-13 |
|  | 23d | Discuss implications of the results for practice, policy, and future research. | Page12-13 |
| **OTHER INFORMATION** | | |  |
| Registration and protocol | 24a | Provide registration information for the review, including register name and registration number, or state that the review was not registered. | Page2 |
|  | 24b | Indicate where the review protocol can be accessed, or state that a protocol was not prepared. | None |
|  | 24c | Describe and explain any amendments to information provided at registration or in the protocol. | None |
| Support | 25 | Describe sources of financial or non-financial support for the review, and the role of the funders or sponsors in the review. | Page14 |
| Competing interests | 26 | Declare any competing interests of review authors. | Page14 |
| Availability of data, code and other materials | 27 | Report which of the following are publicly available and where they can be found: template data collection forms; data extracted from included studies; data used for all analyses; analytic code; any other materials used in the review. | Page 8 |

For more information, visit: <http://www.prisma-statement.org/>

**Search strategy**

MEDLINE via Ovidsp：

1. exp heart failure/

2. ((heart or cardia* or myocardial) adj3 (failure or insufficienc* or decompensat* or dysfunction)).tw.

3. 1 or 2

4. exp Ventricular Function/

5. ((preserved or normal or greater) adj5 (ejection fraction or cardiac function or EF or LVEF)).tw.

6. (preserved systolic function or normal systolic function or HFpEF or HF-pEF or HFnEF or HF-nEF or DHF or diastolic*).tw.

7. 4 or 5 or 6

8. 3 and 7

9. exp kidney disease/

10. exp kidney failure/

11. exp chronic kidney failure/

12. exp renal replacement therapy/

13. exp hemodialysis/

14. exp uremia/

15. (end-stage renal or end-stage kidney or end stage renal or end stage kidney).tw.

16. (chronic kidney or chronic renal).tw.

17. dialysis.tw.

18. (hemodialysis or haemodialysis).tw.

19. (hemofiltration or haemofiltration).tw.

20. (hemodiafiltration or haemodiafiltration).tw.

21. (kidney disease* or renal disease* or kidney failure or renal failure).tw.

22. (ESRF or ESKF or ESRD or ESKD).tw.

23. (CKF or CKD or CRF or CRD).tw.

24. (PD or CAPD or CCPD or APD).tw.

25. (predialysis or pre-dialysis).tw.

26. ur?emi$.tw.

27. or/9-26

28. 8 and 27

29. limit 28 to human

EMBASE via Ovidsp：

1. exp heart failure/

2. ((heart or cardia* or myocardial) adj3 (failure or insufficienc* or decompensat* or dysfunction)).tw.

3. 1 or 2

# 4. exp Ventricular Function/

5. ((preserved or normal or greater) adj5 (ejection fraction or cardiac function or EF or LVEF)).tw.

6. (preserved systolic function or normal systolic function or HFpEF or HF-pEF or HFnEF or HF-nEF or DHF or diastolic*).tw.

7. 4 or 5 or 6

8. 3 and 7

9. exp kidney disease/

10. exp kidney failure/

11. exp chronic kidney failure/

12. exp renal replacement therapy/

13. exp hemodialysis/

14. exp uremia/

15. (end-stage renal or end-stage kidney or end stage renal or end stage kidney).tw.

16. (chronic kidney or chronic renal).tw.

17. dialysis.tw.

18. (hemodialysis or haemodialysis).tw.

19. (hemofiltration or haemofiltration).tw.

20. (hemodiafiltration or haemodiafiltration).tw.

21. (kidney disease* or renal disease* or kidney failure or renal failure).tw.

22. (ESRF or ESKF or ESRD or ESKD).tw.

23. (CKF or CKD or CRF or CRD).tw.

24. (PD or CAPD or CCPD or APD).tw.

25. (predialysis or pre-dialysis).tw.

26. ur?emi$.tw.

27. or/9-26

28. 8 and 27

29. limit 28 to human

Cochrane Library：

1. MeSH descriptor: [Heart Failure] explode all trees

2. ((heart or cardia* or myocardial) near/3 (failure or insuHicienc* or decompensat*)):ti,ab,kw

3. #1 or #2

4. MeSH descriptor: [Ventricular Dysfunction] explode all trees

5. MeSH descriptor: [Ventricular Function] explode all trees

6. ((preserved or normal or greater) near/5 ("ejection fraction" or "cardiac function" or "EF" or "LVEF")):ti,ab,kw (Word variations have been searched

7. ("preserved systolic function" or "normal systolic function" or "HFpEF" or "HF-pEF" or "HFnEF" or "HF-nEF" or "DHF" or diastolic*):ti,ab,kw

8. #4 or #5 or #6 or #7

9. #3 and #8

10. MeSH descriptor: [Kidney Diseases] explode all trees

11. MeSH descriptor: [Renal Insufficiency] explode all trees

12. MeSH descriptor: [Renal Insufficiency, Chronic] explode all trees

13. MeSH descriptor: [Kidney Failure, Chronic] explode all trees

14. MeSH descriptor: [Renal Replacement Therapy] explode all trees

15. MeSH descriptor: [Renal Dialysis] explode all trees

16. MeSH descriptor: [Uremia] explode all trees

17. ("end-stage renal" or "end-stage kidney" or "end stage renal" or "end stage kidney"):ti,ab,kw

18. ("chronic kidney" or "chronic renal"):ti,ab,kw

19. (dialysis):ti,ab,kw

20. (hemodialysis or haemodialysis):ti,ab,kw

21. (hemofiltration or haemofiltration):ti,ab,kw

22. (hemodiafiltration or haemodiafiltration):ti,ab,kw

23. ("kidney disease*" or "renal disease*" or "kidney failure" or "renal failure"):ti,ab,kw

24. (ESRF or ESKF or ESRD or ESKD):ti,ab,kw

25. (CKF or CKD or CRF or CRD):ti,ab,kw

26. (PD or CAPD or CCPD or APD):ti,ab,kw

27. (predialysis or pre-dialysis):ti,ab,kw

28. (ur*emi*):ti,ab,kw

29. #10 or #11 or #12 or #13 or #14 or #15 or #16 or #17 or #18 or #19 or #20 or #21 or #22 or #23 or #24 or #25 or #26 or #27 or #28

30. #9 and #29

Table S1 Risk of bias graph for included retrospective cohort study accessed by ROBINS-I

| Study | Bias due to confounding | Bias in selection of participants into the study | Bias in classification of interventions | Bias due to deviations from intended interventions | Bias due to missing data | Bias in measurement of outcomes   | Bias in selection of the reported result | Overall bias |
| --- | --- | --- | --- | --- | --- | --- | --- | --- |
| Ahmed 2013 | Low | Low | Low | Moderate | Low | Low | Low | Moderate |
| Gurwitz 2017 | Low | Low | Low | Low | Low | Low | Low | Low |
| Tsujimoto 2018 | Low | Low | Low | Low | Low | Low | Low | Low |


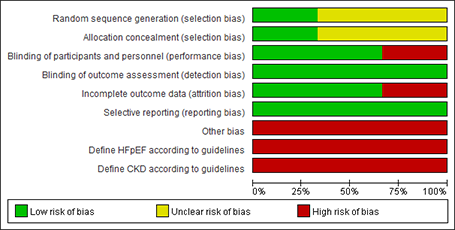


**Fig S1** Risk of bias graph for included randomized controlled study


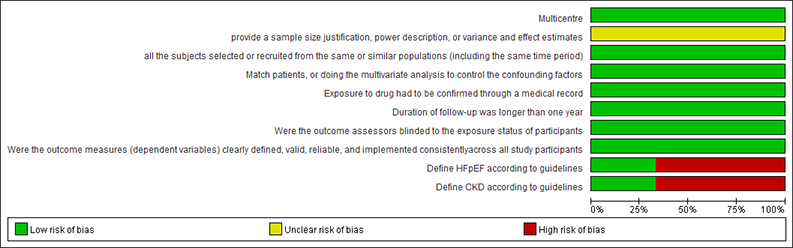
**Fig S2** Risk of bias graph for included retrospective cohort study accessed by NOS and NHLBI


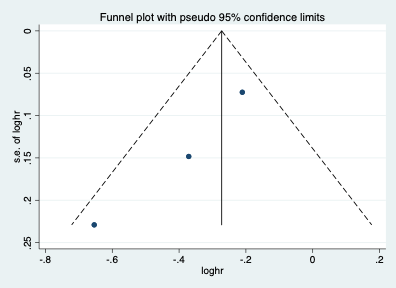


**Fig S3** Funnel plot on all-cause mortality


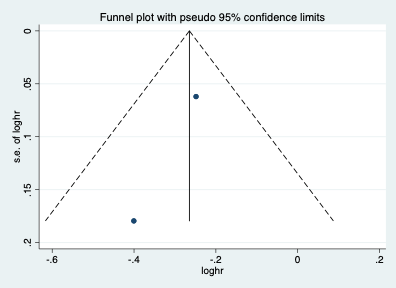


**Fig S4** Funnel plot on all-cause hospitalization


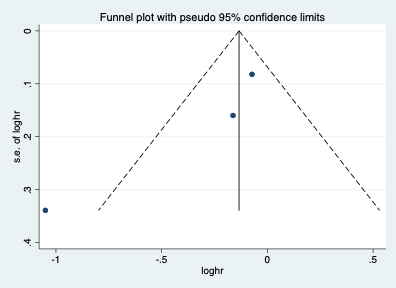


**Fig S5** Funnel plot on hospitalization for heart failure
